# Ten priorities that are shaping the future of mental health prevention

**DOI:** 10.1007/s44192-025-00290-7

**Published:** 2025-09-30

**Authors:** Marco Colizzi

**Affiliations:** 1 Unit of Psychiatry and Eating Disorders, Department of Medicine (DMED), University of Udine, Udine, 33100 Italy; 2 Department of Psychosis Studies, Institute of Psychiatry, Psychology and Neuroscience, King’s College London, London, SE5 8AF UK

## Abstract

Mental health is an escalating global health priority, yet prevention strategies remain underdeveloped and underutilized. This commentary outlines ten priorities to advance a modern, equitable, and effective approach to mental health prevention. It advocates expanding the At-Risk Mental State (ARMS) framework beyond psychosis to include other common mental disorders, and calls for routine, dynamic risk assessment, particularly targeting early-life adversities. A transdiagnostic approach is encouraged to better identify and respond to nuanced, dimensional early signs of psychological distress. Emphasizing a neurodevelopmental perspective, the commentary supports life-course interventions and improved continuity between child and adult mental health services. It urges to redesign mental health systems to enable early access and sustained youth engagement, particularly through multidisciplinary, integrated care models that encompass both mental health and addiction services. The commentary also highlights growing evidence of shared biological mechanisms, such as inflammation and metabolic dysregulation, linking mental and physical health, reinforcing the need for holistic prevention strategies. Finally, it underscores the necessity of societal and policy-level interventions to address structural determinants of mental illness, including inequality, environmental stressors, and youth marginalization. Together, these priorities present a proactive and collaborative vision of prevention that spans individual, community, and systemic levels. Achieving this vision requires a fundamental shift from reactive clinical care to preventive, intersectoral public health action. While ambitious, such a transformation is essential to reduce the global burden of mental illness and promote lifelong mental well-being.

## Introduction

The World Health Organization advocates for population-level health interventions to be guided by a health-promoting perspective [1], a principle that applies equally to mental health. Yet, we remain far from delivering interventions that genuinely empower individuals and communities to manage their mental health and address its underlying determinants. According to recent meta-analytic evidence, pooled prevalence of unmet need for mental health care among adolescents is 54.0%, with mental health literacy, preventive interventions, and mental health promotion representing key factor for mental health improvement [2]. Why, then, does prevention continue to be one of the most underdeveloped areas in mental healthcare? This commentary presents ten key priorities to inform a modern, equitable, and effective approach to mental health prevention.

## Prioritize the At-Risk mental state (ARMS)

The concept of preventing psychotic disorders by identifying high-risk individuals during the early stages of the pathological process will be a century old in 2027 [3]. This paradigm was refined in the 1990s, with the realization that, even if it was not always possible to completely prevent a psychotic episode, it was certainly feasible to minimize its impact on functioning and long-term disability through early intervention strategies [4]. So, where do we stand today? While we have made progress in developing services and diagnostic tools for psychosis [5], it has also become clear that the At-Risk Mental State (ARMS) concept has yet to be fully extended to other common mental disorders such as depression, anxiety, substance abuse, and eating disorders [6]. This understanding follows evidence that most ARMS individuals do not transition to psychosis but rather develop persistent mood, anxiety, or personality disorders [7]. For instance, a recent study aimed to prospectively validate the Clinical High At Risk Mental State (CHARMS) criteria for identifying young people at risk of imminent progression to a severe mental disorder. The CHARMS + group showed higher transition rates, more severe symptoms, and greater treatment needs than the CHARMS − group. Baseline depressive symptom severity was the main predictor of transition at 12 months. Meeting criteria for three or more at-risk subgroups significantly increased transition risk (> 50%). Among those who clinically transitioned, 61% met multiple at-risk criteria at baseline, suggesting that complex symptom profiles (e.g., comorbidities) are key risk indicators. High comorbidity and diffuse symptoms were common, with 24% of CHARMS + participants meeting criteria for 3–4 risk groups [8]. Consistently, independent research suggests that similar risk factors may contribute to a range of psychopathologies, with early indicators often being dimensional rather than categorical [9].

## Adopt a transdiagnostic approach

Although homotypic prediction models (e.g., a disorder predicting itself over time) have their value, heterotypic prediction models (e.g., one disorder predicting another) better reflect real-world clinical experiences. Clinicians often encounter nuanced phenotypes. There is robust evidence of cross-prediction between anxiety and depression during the transition from adolescence to adulthood. Other conditions, such as substance use disorders, can fully account for the homotypic prediction pattern of depression over time. Internalizing and externalizing disorders also predict psychotic-spectrum symptoms, and vice versa [10]. Adopting a transdiagnostic ARMS paradigm could allow us to detect a broader range of high-risk individuals, such as those presenting with sub-threshold depressive, bipolar, or borderline symptoms, rather than focusing solely on the emergence of positive psychotic symptoms [11]. Disorder-specific diagnostic approaches may obscure shared risk factors and symptom dimensions and are particularly difficult to apply to children and adolescents, where disorders often begin but are preceded by non-specific emotional, cognitive, or behavioural disturbances that do not fit neatly into a single diagnostic category [12]. Thus, a transdiagnostic approach may help in understanding both the underlying mechanisms and observable symptoms that span traditional diagnostic categories. At the same time, prevention efforts must account for neurodevelopment, which extends across the entire high-risk age window for the onset of mental disorders.

## Make risk assessment routine

Mental health prevention strategies must include dynamic risk assessment tools capable of identifying individuals entering a state of risk. But which risk factors should be monitored, when, and for which clinical conditions? While c*ondition-specific markers* (e.g., attenuated psychotic symptoms for psychosis, anhedonia for depression) are still important, *transdiagnostic factors* such as persistent subthreshold symptoms (e.g., mood instability, anxiety), family history of mental disorder and other parental factors, trauma or chronic stress exposure, functional decline, cognitive errors or emotional dysregulation and sleep disturbances, should be monitored. Ideally, monitoring should be on an ongoing and adaptive basis, rather than a one-time screening, and cover developmentally sensitive periods, such as adolescence and early adulthood, after major life stressors, and following initial symptom onset of decline in functioning [13]. As an example, a global survey of 51,945 adults in 21 countries found that childhood adversities account for 29.8% of all mental disorders, with limited specificity across conditions. Strikingly, the population-attributable risk proportions (PARPs) suggest that eliminating childhood adversities could prevent up to 38.2% of disorders when focusing on childhood-onset cases [14]. Further evidence highlights that the earlier risk and protective factors emerge, the more significant their impact on mental health [15]. This underscores the importance of conducting risk assessments as early as possible - ideally during childhood or adolescence - when early intervention can be most effective in preventing or mitigating long-term psychological consequences [16, 17].

## Embrace the neurodevelopmental perspective

A growing body of evidence indicates that mental disorders are rooted in early neurodevelopmental trajectories. For example, the neurodevelopmental hypothesis of schizophrenia suggests that although the condition typically emerges in adolescence or early adulthood, it is largely influenced by earlier developmental events [18]. Moreover, genetic overlaps exist between schizophrenia and other neurodevelopmental disorders such as autism spectrum disorder, intellectual disability, and ADHD [19]. These findings challenge the validity of current diagnostic categories, as they are affected by both horizontal comorbidities (e.g., two disorders peaking in the same age window) and vertical comorbidities (e.g., adult-onset disorders linked to child-onset conditions via shared developmental roots). The neurodevelopmental gradient hypothesis has integrated these insights, suggesting that mental disorders can be ranked based on the severity of neurodevelopmental impairment, with implications for age of onset, treatment timing, and outcomes [20]. Emerging evidence suggests that not only severe mood disorders or childhood-onset conditions, but also more common and potentially less severe conditions - such as depression and personality traits - may lie along a neurodevelopmental continuum, further emphasizing the lasting impact of early-life experiences on mental health trajectories across the lifespan [21]. This perspective supports the implementation of tiered, developmentally informed prevention strategies, beginning with early screening for neurodevelopmental concerns, ideally starting in infancy or early childhood. Early identification of at-risk individuals would enable mental health services to adopt a proactive, stepped-care model, in which interventions are tailored to developmental stage and risk severity.

## Redesign services for early access and engagement

We need to act more swiftly and effectively. The average delay between symptom onset and intervention for any mental disorder, known as the duration of untreated illness, is 8 to 10 years, with individuals aged 0–25 experiencing the greatest delays [22]. Young people, particularly males, those from socioeconomically disadvantaged backgrounds, and ethnic minorities, are less likely to access mental health services, often due to stigma and disengagement [23]. Overall, current services struggle to promptly deliver specialist care to youth populations [24]. When treatment is provided, it is frequently not guided by evidence-based protocols [25]. Many services remain crisis-driven [26], with emergency mental health presentations among children and adolescents not uncommon [27]. Although deinstitutionalization policies have made some progress, there is significant global variability in the implementation of community-based mental health services, particularly for youth [28, 29]. Mental health services must therefore be redesigned to facilitate early access through low-threshold entry points (e.g., schools, community centres) and user-friendly formats (e.g., digital platforms). Co-designing services with youth and caregivers can further improve engagement and effectiveness [30]. Tailored interventions often require a multidisciplinary mix of services.

## Ensure continuity between child and adult services

The risk of developing a mental disorder peaks between adolescence and young adulthood [31], a period characterized by significant stressors such as physical changes, starting college, and increasing independence [32]. During this time, some individuals may transition from child to adult mental health services. However, this transition is often perceived as arbitrary and disruptive, complicating the management of clinical conditions and life events [33]. The current divide between child and adult psychiatric services often fails to ensure continuity of care and does not always address the specific developmental needs of young people [34]. Key challenges include the need for flexible transition periods, potential disengagement from care, loss of motivation, and the difficulty of replacing established therapeutic relationships. Some researchers advocate for dedicated transition services that provide overlap and coordination between child and adult services, promote active involvement in the transition process, empower young people, and reduce dropouts [32]. A recently developed approach establishes integrated, youth-friendly mental health services for transition-age individuals, typically 16–25 years, bridging child and adult care through accessible, non-stigmatizing support. Multidisciplinary teams offer early intervention and tailored care for a broad range of mental health challenges, including neurodevelopmental disorders. Early evidence shows these services improve engagement, reduce hospitalizations, and enhance outcomes by providing timely, coordinated care during this critical period [32].

## Promote integrated and coordinated mental health supports

No single profession possesses all the tools required for effective prevention. True multidisciplinary collaboration is essential for comprehensive assessment of biological, psychological, and social risk factors, as well as for the delivery of personalized, integrated interventions. Depending on the individual’s presenting problems, whether mental, behavioural, situational, physical, sexual, vocational, or substance-related, the type of service provided, the primary provider, and the funding source may all vary [35]. Various global models have successfully implemented integrated, multidisciplinary care systems through extended primary care networks [36]. This approach involves both vertical and horizontal integration. Vertically, primary care services can escalate to more specialized tiers, from intermediate and secondary care to tertiary care. Horizontally, each tier benefits from coordinated services, including community health and wellbeing initiatives, mental health triage systems, acute community services, clinical networks, and specialized mental, physical, and disability services [12]. Both coordinated and integrated approaches can be used, as they are not mutually exclusive but rather complementary in addressing complex community needs.

## Integrate addiction and mental health prevention

Substance use is preventable, but its conceptualization as a disease immediately raises the question of who is responsible for treating it, especially in an era when medicine has become so broad and complex that no single individual can comprehensively master it, and specialized expertise is required across various domains. A growing body of evidence highlights that the global rise in mental disorders among young people has occurred in parallel with increased recreational drug use [37] and a heightened societal interest in substances [38]. Therefore, no effective mental health prevention strategy can overlook the harmful impact of substance use [39]. Moreover, individuals with substance use disorders often present with comorbid physical health conditions, such as chronic pain [40] and cardiovascular disease [41]. Both psychiatric disorders [42] and substance use disorders [41] are associated with an elevated risk of cardiometabolic diseases, further underscoring the need for a paradigmatic shift in how these interconnected issues are addressed. Recent evidence emphasizes the limitations of *serial care* - an approach that, for instance, requires patients to achieve complete abstinence or undergo detoxification before addressing co-occurring psychiatric conditions such as depression or anxiety. This method can significantly delay necessary mental health treatment and reduce overall effectiveness. Equally inadequate is the assumption - often described as a “Trojan Horse” approach - that treating psychiatric symptoms alone will indirectly resolve substance use disorders. In contrast, *integrated care*, which addresses both substance use and mental health conditions simultaneously, has proven more effective. This model also typically extends beyond clinical treatment, incorporating social supports such as housing, employment, and financial assistance, reinforcing the need for holistic, individualized care strategies [43].

## Foster collaboration with other medical disciplines

Our understanding of the brain basis of mental illness has evolved over the past three and a half millennia, with remarkable acceleration in recent decades [44]. As neuroscience and psychiatry converge more closely than ever in the current century, psychiatric knowledge is increasingly expanding beyond the brain. The roles of the gut microbiome, metabolites, omics, hormones, and stress in shaping various health conditions, including mental disorders, are now widely recognized [45]. Consequently, it is not surprising that mental and physical illnesses often share common neurobiological underpinnings. For example, inflammatory processes are known to signal the brain to initiate “sickness behaviour”, an adaptive response to illness characterized by symptoms such as loss of appetite, fatigue, and social withdrawal, which closely mirrors core features of depression and contributes to increased feelings of social disconnection [46]. Furthermore, individuals with co-occurring mental and physical disorders face a significantly reduced life expectancy, underscoring the urgent need for prevention and early detection of comorbidities to reduce premature mortality in these populations [47]. Greater attention must be directed toward individuals at risk of these pathological processes, their biological underpinnings, and the development of treatments targeting inflammation and metabolic dysregulation. Achieving this will require sustained collaboration of psychiatry within the broader medical field, fostering a truly bidirectional and interdisciplinary approach to care.

## Invest in societal and policy-level interventions

Advancements in healthcare and nutrition, extended access to education, and the widespread adoption of new technologies have created the potential for today’s adolescents to be the healthiest generation in history [48]. Yet, the *Lancet Psychiatry Commission on youth mental health* has recently reported a steady decline in the mental health of emerging adults over the past two decades, likely driven by new and evolving threats to adolescent well-being. These include the increased availability of unhealthy products, social media and technological changes, academic and labor market challenges, declining family stability, environmental degradation, armed conflict, and mass migration, trends that were further exacerbated by the COVID-19 pandemic [49]. This global megatrend underscores the profound impact of societal changes on youth mental health and highlights the urgent need for prevention strategies that reach beyond clinical settings. Effective responses must involve advocacy for policies that address structural inequalities and promote mental well-being at the population level [12]. Supporting adolescents is not only critical for ensuring their long-term psychophysical health but also yields intergenerational benefits, as healthier young people are more likely to become healthier parents, positively influencing the well-being of the next generation [50]. Prevention strategies should therefore comprehensively address children’s physical (e.g., healthy nutrition), psychological (e.g., resiliency), and social (e.g., supportive environments) needs to foster brain development, emotional regulation, and cognitive functioning. These interventions can positively modulate genetic vulnerabilities [51] and stress responses [52]. Otherwise, early adversities such as neglect, trauma, and inadequate care may have lasting negative consequences on education, psychosocial skills, physical health, and aging trajectories [53].

## Limitations and barriers

While the proposed strategies offer a strong framework for improving mental health care, significant barriers may hinder their implementation. These include high costs, entrenched institutional interests, disparities in access, and the complexity of engaging stakeholders across disciplines and levels. For instance, evidence shows that screening and physician notification alone are not enough to prompt adequate depression treatment [54]. Care quality is often shaped more by healthcare system characteristics than by individual clinical profiles, with financial barriers posing greater challenges than stigma. However, quality improvement (QI) programs in managed primary care that expand access to depression treatment - without making it mandatory - have been shown to improve care quality, mental health outcomes, and employment retention without increasing overall medical visits [55]. These findings highlight the need for structural reforms and system-level strategies that address real-world implementation challenges.

## Conclusions

Mental health professionals alone cannot meet the growing demand for mental health prevention. To address this challenge effectively, they must shift from working in isolation to engaging in collaborative, system-level approaches, from reactive care to proactive, early intervention, and from clinical settings to broader community and policy arenas. In this expanded role, they carry the responsibility of advancing scientific knowledge, fostering ethical discourse, and guiding health institutions and governments toward policies that prioritize mental well-being at the societal level. While effective mental health prevention is an ambitious and complex goal, it is achievable, provided it is systematically integrated across all stages of life. The task is undeniably challenging, but the potential benefits make it not only worthwhile, but essential.

## Data Availability

No datasets were generated or analysed during the current study.
